# Cumulative estimated glucose disposal rate predicts frailty progression in Chinese adults with diabetes: a 9-year follow-up cohort study

**DOI:** 10.3389/fmed.2025.1617556

**Published:** 2025-12-22

**Authors:** Bin Zhao, Zhian Liang, Xiaojie Zhao, Xiangchen Dai

**Affiliations:** Department of Vascular Surgery, Tianjin Medical University General Hospital, Tianjin, China

**Keywords:** diabetes mellitus, frailty, insulin resistance, estimated glucose disposal rate, longitudinal study, cohort study

## Abstract

**Introduction:**

Frailty is a common adverse outcome in diabetes that is linked to reduced quality of life and increased mortality and is strongly associated with insulin resistance. However, the relationship between long-term cumulative insulin resistance and frailty progression remains unclear, particularly in Chinese adults with diabetes.

**Methods:**

We used data from the China Health and Retirement Longitudinal Study. We included 451 participants aged 45 years or older with baseline diabetes in 2011 who had frailty index scores based on 26 health deficit items across four waves (2011, 2015, 2018, 2020) and complete data in 2011 and 2015 to calculate cumulative estimated glucose disposal rate, cumulative triglyceride–glucose index, and cumulative triglyceride–glucose–body mass index. Linear mixed-effects models with random intercepts, adjusted for baseline age, sex, smoking, alcohol consumption, baseline estimated glucose disposal rate, and cardiovascular disease history, were used to examine the interaction between standardized cumulative indices and time in years on frailty index trajectories. Prespecified subgroup interactions were also evaluated.

**Results:**

Only the interaction between cumulative estimated glucose disposal rate and time was statistically significant. A higher cumulative estimated glucose disposal rate was associated with a slower annual increase in frailty index (interaction beta = –0.0019 per year, 95% confidence interval –0.0035 to –0.0002, *P* = 0.028), whereas cumulative triglyceride–glucose index and cumulative triglyceride–glucose–body mass index were not associated with the rate of frailty change. Subgroup analyses did not identify statistically significant effect modification by sex, age group, baseline frailty level, baseline cardiovascular disease, residence, or education.

**Discussion:**

Higher cumulative estimated glucose disposal rate, reflecting better sustained insulin sensitivity over four years, was associated with a slower nine-year frailty progression among middle-aged and older Chinese adults with diabetes. Although the effect size is modest and causality cannot be inferred, cumulative estimated glucose disposal rate may serve as a useful indicator for long-term risk assessment and longitudinal monitoring of frailty.

## 1 Introduction

In the context of accelerating global population aging and changing lifestyle patterns, diabetes mellitus is a major public health challenge, particularly in China, where its prevalence continues to rise. Epidemiological data indicate a substantial diabetes burden in China, with prevalence rates exceeding 15% among adults aged 40 years and above in certain regions (1), and often manifesting at a younger age and lower body mass index (BMI) compared to European populations, reflecting unique ethnic and environmental factors (2). Diabetes markedly increases the risk not only for cardiovascular diseases (CVD), including coronary heart disease, stroke, and peripheral artery disease (PAD) (3, 4), but also is strongly associated with various other age-related health issues, among which frailty is gaining attention (5).

Frailty, a clinical syndrome characterized by diminished physiological reserve and heightened vulnerability to stressors, predicts falls, hospitalization, disability, reduced quality of life, and mortality (6, 7). The burden of frailty is particularly pronounced in individuals with diabetes; a recent meta-analysis reported a pooled frailty prevalence of 30.0% among older adults with diabetes, with pre-frailty affecting an additional 45.1% (8). Furthermore, robust evidence links frailty to significantly elevated risks of mortality (HR 1.91), hospitalization (HR 2.19), and disability (HR 3.84) within this vulnerable population (7).

The presence of frailty poses considerable challenges for diabetic patients undergoing surgical interventions, especially within vascular surgery. Vascular procedures, often targeting conditions like PAD, are common, yet concurrent frailty significantly increases the risk of perioperative complications and mortality (9, 10). Frailty indices incorporating diabetes, such as the modified 5-item frailty index (mFI-5), have demonstrated utility in predicting adverse outcomes across various surgical settings (11). Consequently, elucidating the mechanisms underlying frailty development in diabetes and identifying modifiable risk factors are paramount for improving patient prognosis and informing clinical practice, particularly for perioperative risk management and intervention strategies.

IR, the core pathophysiological defect in type 2 diabetes, is widely recognized as a crucial link between metabolic dysregulation and frailty (5, 12). Evidence from large cohort studies, such as the Cardiovascular Health Study, indicates that IR, assessed via homeostasis model assessment of insulin resistance (HOMA-IR), is associated with incident frailty (6). IR likely promotes frailty through multifaceted pathways, including the induction of chronic low-grade inflammation (“inflammaging”), oxidative stress, endothelial dysfunction, and disruption of muscle protein metabolism leading to sarcopenia (13–15), with inflammation and oxidative stress considered common ground for both frailty and metabolic syndromes (5). However, prior research investigating the IR-frailty relationship faces limitations. Many studies employ cross-sectional designs (16), precluding causal inference. Longitudinal studies often focus on the impact of baseline IR levels on incident frailty risk, rarely examining the long-term effects of cumulative IR exposure or dynamic IR changes on the trajectory of frailty progression. Moreover, the gold-standard method for IR assessment (hyperinsulinemic-euglycemic clamp) is impractical for large-scale studies, while common surrogates like HOMA-IR require insulin measurements (17). The eGDR, an IR assessment model based on non-insulin-dependent measures (waist circumference, hypertension status, HbA1c), shows good concordance with clamp techniques and offers feasibility for cohort studies (6, 17). Although baseline eGDR has been linked to CVD events, mortality, and frailty risk (7), research utilizing Cum-eGDR—a potentially better reflection of long-term average IR exposure—to evaluate its impact on the longitudinal rate of frailty progression remains scarce, particularly within the unique context of the Chinese diabetic population.

The CHARLS provides an invaluable resource, featuring a nationally representative sample of middle-aged and older adults, longitudinal follow-up, and comprehensive data collection including physical examinations, questionnaires, and biospecimen analysis (2). Therefore, this study leveraged the CHARLS cohort to prospectively investigate the association between Cum-eGDR over 4 years (W1–W3) is associated with a slower frailty FI change trajectory over up to 9 years (Wave 1–Wave 5). We also compared Cum-eGDR with Cum-TyG and Cum-TyG-BMI and examined effect modification by sex, age, baseline frailty, baseline CVD history, residence, and education. We hypothesized that higher Cum-eGDR, indicating better average insulin sensitivity, would be associated with a slower rate of frailty progression.

## 2 Materials and methods

### 2.1 Data source and study design

This study utilized data from the CHARLS, a nationally representative longitudinal cohort of middle-aged and older Chinese adults (aged ≥ 45 years) collecting social, economic, and health information. The CHARLS design adheres to strict ethical standards and received approval from the Institutional Review Board of Peking University (IRB00001052-11015). All participants provided written informed consent at baseline. We analyzed data from four CHARLS waves: 2011 (Wave 1, baseline), 2015 (Wave 3), 2018 (Wave 4), and 2020 (Wave 5).

### 2.2 Study population

**FIGURE 1:**
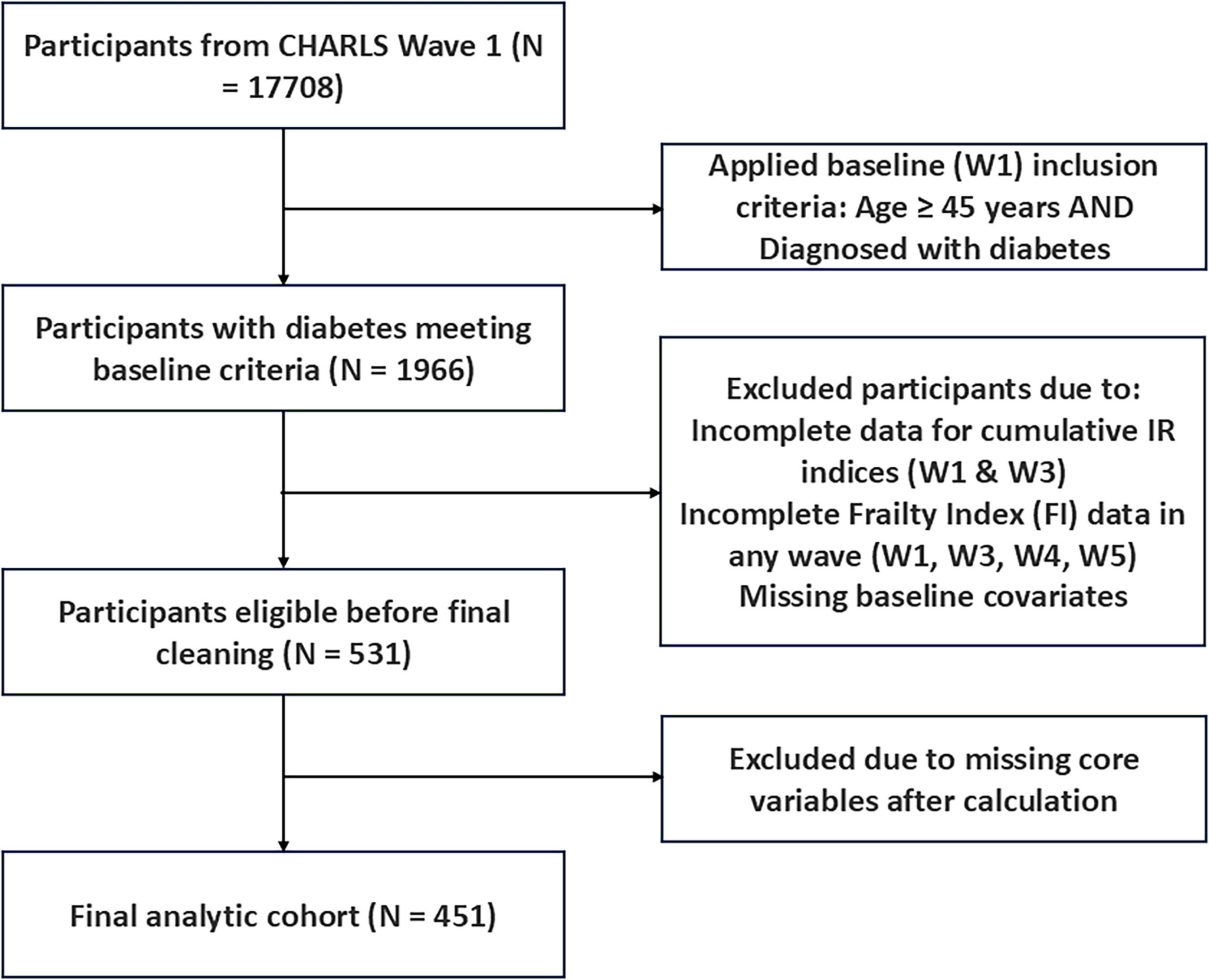
Flowchart of participant selection. From CHARLS Wave 1 (*N* = 17,708), participants with diabetes meeting baseline criteria (*N* = 1,966) were identified. Exclusions included incomplete data for cumulative IR indices (W1 & W3), incomplete FI data in any wave (W1, W3, W4, W5), and missing baseline covariates, followed by final cleaning for missing core variables after calculation. The final analytic cohort comprised *N* = 451. IR, insulin resistance; FI, Frailty Index; W, wave.

Participants from the CHARLS baseline survey (Wave 1) were initially considered. Inclusion criteria were: (1) age ≥ 45 years at baseline; (2) baseline diabetes according to CHARLS criteria (self-report, medication use, fasting glucose, or HbA1c; variable *diabe (W1)*); (3) complete data available at both Wave 1 and Wave 3 necessary for calculating cumulative IR indices (eGDR, TyG, TyG-BMI); (4) valid FI assessment data available for Wave 1, 3, 4, and 5, allowing ≤ 20% missing FI components per wave; (5) complete baseline covariate information. Individuals without diabetes at baseline or those with loss to follow-up or essential longitudinal data missing were excluded. After applying these criteria, the final analytic cohort comprised 451 participants with baseline diabetes (Figure 1).

### 2.3 Exposure assessment

#### 2.3.1 IR surrogate calculation (Wave 1 and Wave 3)

Three surrogate indices for IR were calculated at baseline (W1) and follow-up (W3). The eGDR was calculated using the formula: eGDR (mg/kg/min) = 21.158−0.09 × waist circumference (cm) − 3.407 × hypertension (1/0) − 0.551 × HbA1c (%), where hypertension status [*hibpe* (*W1*)] was defined based on self-report, medication use, or measured blood pressure (systolic ≥ 140 mmHg or diastolic ≥ 90 mmHg), and HbA1c (*bl_hbalc*) and waist circumference (*mwaist*) were obtained from biospecimen analysis and physical examination, respectively (18). The TyG was calculated as TyG = ln [(fasting triglycerides, mg/dL) × (fasting glucose, mg/dL)/2], using fasting triglycerides (*bl_tg*) and glucose (*bl_glu*) measured from venous blood samples (units confirmed as mg/dL). The Triglyceride-Glucose-Body Mass Index (TyG-BMI) was calculated as TyG-BMI = TyG × BMI (kg/m^2^), where BMI (*bmi*) was derived from measured height and weight.

#### 2.3.2 Cumulative IR indices (wave 1—wave 3)

To reflect the cumulative exposure from Wave 1 (W1) to Wave 3 (W3), the area under the curve (AUC) was estimated using the trapezoidal rule. Wave 2 (2013) lacked consistently available and harmonized biospecimen and anthropometric inputs, such as HbA1c, waist circumference, fasting glucose, and triglycerides, that were required to compute IR surrogates; therefore, cumulative IR indices were defined over W1–W3. With two time points, the AUC reduces to a time-weighted average:

Cumulative Index_*W1*–*W3*_ = $\left(\frac{\mathrm{IndexW1}+\mathrm{IndexW3}}{2}\right)\times \left(t\,W\,3-t\,W\,1\right)$ where Index denotes eGDR, TyG, or TyG-BMI, and (*tW*3-*tW*1) = 4 years by study design. All cumulative indices and other continuous predictors (such as baseline age, baseline eGDR) were standardized to Z-scores prior to modeling.

### 2.4 Outcome assessment: frailty index

The primary outcome was the longitudinal trajectory of the FI assessed at Waves 1, 3, 4, and 5. The FI was constructed based on the cumulative health deficit model (19). Following established methods and CHARLS data availability, we included 26 health-deficit items (excluding BMI and grip strength due to missingness in later waves) covering chronic diseases, symptoms/signs, sensory impairments, ADL/IADL, cognition, and mood. A detailed list of the FI components and their coding criteria (defining a deficit as“1”) is provided in Supplementary Table 1. For each participant at each wave, the FI score was calculated as the number of deficits present divided by the total number of non-missing deficits assessed for that individual at that wave. If more than 20% of the FI components were missing for an individual at a specific wave, the FI score for that wave was treated as missing.

### 2.5 Covariate assessment

Baseline (Wave 1) covariates were obtained through standardized questionnaires and physical examinations, including: age [*age(W1)*], continuous), sex [*gender (W1)*, 0 = Female, 1 = Male], smoking status [*smoken (W1)*, 0 = Never smoked, 1 = Ever/Current smoker], alcohol consumption [*drink l(W1)*, 0 = Never/Not current drinker, 1 = Current drinker], baseline CVD history (*Baseline-CVD*, defined as self-reported heart disease [*hearte (W1)* = 1] or stroke [*stroke (W1)* = 1], baseline eGDR [*eGDR (W1)*, continuous], education level [*edu (W1)*, categorized as 1 = Illiterate/Primary, 2 = Middle school, 3 = High school, 4 = College or above], and residence [*rura l(W1)*, 0 = Urban, 1 = Rural]. Terminology was harmonized to use hyphenated forms (such as “Cum-eGDR”) throughout.

### 2.6 Statistical analysis

Baseline characteristics of the final study population (*N* = 451) were described using means ± standard deviations (SD) or frequencies (percentages) and were compared across quartiles of Cum-eGDR (W1–W3) using ANOVA, Kruskal-Wallis, or Chi-squared tests, as appropriate. To assess the longitudinal association between IR indices and the FI trajectory, we employed LME with the FI score as the dependent variable and a random intercept for each participant ID. Time was modeled as years since baseline (continuous). Each standardized cumulative index (Cum-eGDR, Cum-TyG, and Cum-TyG-BMI) was entered separately with an index × time interaction, adjusting for all baseline covariates. Model fit was compared using Akaike’s Information Criterion (AIC) and Bayesian Information Criterion (BIC). To formally test the primary hypothesis, we compared the best-performing cumulative model with a similarly adjusted model using standardized baseline eGDR (both including index × time). Potential effect modification was explored with three-way interactions (index × time × subgroup) for sex, age group, baseline FI, education, residence, and baseline CVD. All analyses were performed using standard statistical software; two-sided *P* < 0.05 was considered statistically significant.

## 3 Results

### 3.1 Participant selection and baseline characteristics

The participant selection process is detailed in Figure 1. After applying exclusion criteria, a total of 451 individuals with baseline diabetes (in 2011) and complete longitudinal data for FI assessment and cumulative IR index calculation were included in the final analysis. Baseline characteristics (2011) of the study population, stratified by quartiles of Cum-eGDR (W1–W3), are presented in Table 1. The overall cohort had a mean baseline age of 57.55 ± 7.35 years, with 52.8% being male. The mean baseline FI score, calculated based on 26 deficit items, was 0.21 ± 0.09. Significant differences in baseline metabolic and health profiles were observed across Cum-eGDR quartiles. Specifically, participants in the higher Cum-eGDR quartiles (Q4 vs. Q1) exhibited significantly better baseline insulin sensitivity markers [higher *eGDR (W1)*, lower *TyG (W1)* and *TyG-BMI (W1)*; all *P* < 0.001], lower baseline frailty levels [*Fi-score (W1)*; *P* < 0.001], and a significantly lower prevalence of baseline cardiovascular disease (heart disease or stroke; *Baseline-CVD*; *P* < 0.001). Furthermore, a significant difference in urban/rural residence was noted (*P* = 0.014), with a higher proportion of rural residents observed in the highest Cum-eGDR quartile (Q4). No statistically significant differences were found across groups for baseline age, sex distribution, smoking status, or education level.

**TABLE 1:** Baseline characteristics of participants with diabetes according to quartiles of Cum-eGDR (W1–W3).

Characteristic	Overall (*N* = 451)	Q1 (*N* = 113)	Q2 (*N* = 113)	Q3 (*N* = 112)	Q4 (*N* = 113)	*P*-value
Age (W1) [mean (SD)]	57.55 (7.35)	58.52 (7.57)	58.19 (7.00)	56.68 (7.33)	56.81 (7.39)	0.172
eGDR (W1) [mean (SD)]	7.68 (2.38)	5.09 (0.98)	6.59 (0.96)	8.45 (1.38)	10.62 (1.28)	< 0.001
TyG (W1) [mean (SD)]	9.35 (0.87)	9.62 (0.91)	9.48 (0.87)	9.19 (0.81)	9.11 (0.80)	< 0.001
TyG-BMI (W1) [Mean (SD)]	247.31 (206.35)	276.28 (50.33)	244.37 (40.52)	261.56 (405.61)	207.15 (34.22)	< 0.001
Fi-score (W1) [Mean (SD)]	0.21 (0.09)	0.24 (0.09)	0.22 (0.08)	0.21 (0.10)	0.18 (0.07)	< 0.001
Cum-eGDR (W1–W3) [Mean (SD)]	29.16 (8.92)	18.50 (3.07)	25.02 (1.58)	31.83 (2.37)	41.32 (3.74)	< 0.001
Gender (W1) (N, %)						0.175
0	213 (47.2%)	52 (46.0%)	54 (47.8%)	45 (40.2%)	62 (54.9%)	
1	238 (52.8%)	61 (54.0%)	59 (52.2%)	67 (59.8%)	51 (45.1%)	
Smoken (W1) (N, %)						0.673
0	308 (68.3%)	80 (70.8%)	80 (70.8%)	72 (64.3%)	76 (67.3%)	
1	143 (31.7%)	33 (29.2%)	33 (29.2%)	40 (35.7%)	37 (32.7%)	
Drinkl (W1) (N, %)						0.056
0	284 (63.0%)	80 (70.8%)	74 (65.5%)	60 (53.6%)	70 (61.9%)	
1	167 (37.0%)	33 (29.2%)	39 (34.5%)	52 (46.4%)	43 (38.1%)	
Hearte (W1) (N, %)						0.002
0	375 (83.1%)	85 (75.2%)	88 (77.9%)	99 (88.4%)	103 (91.2%)	
1	76 (16.9%)	28 (24.8%)	25 (22.1%)	13 (11.6%)	10 (8.8%)	
Stroke (W1) (N, %)						0.05
0	438 (97.1%)	106 (93.8%)	109 (96.5%)	111 (99.1%)	112 (99.1%)	
1	13 (2.9%)	7 (6.2%)	4 (3.5%)	1 (0.9%)	1 (0.9%)	
Edu (W1) (N, %)						0.974
1	153 (33.9%)	42 (37.2%)	36 (31.9%)	37 (33.0%)	38 (33.6%)	
2	115 (25.5%)	29 (25.7%)	33 (29.2%)	27 (24.1%)	26 (23.0%)	
3	128 (28.4%)	29 (25.7%)	32 (28.3%)	32 (28.6%)	35 (31.0%)	
4	55 (12.2%)	13 (11.5%)	12 (10.6%)	16 (14.3%)	14 (12.4%)	
Baseline-CVD (N, %)						< 0.001
0	364 (80.7%)	79 (69.9%)	85 (75.2%)	98 (87.5%)	102 (90.3%)	
1	87 (19.3%)	34 (30.1%)	28 (24.8%)	14 (12.5%)	11 (9.7%)	

### 3.2 Model comparisons to identify the optimal predictive approach

**FIGURE 2:**
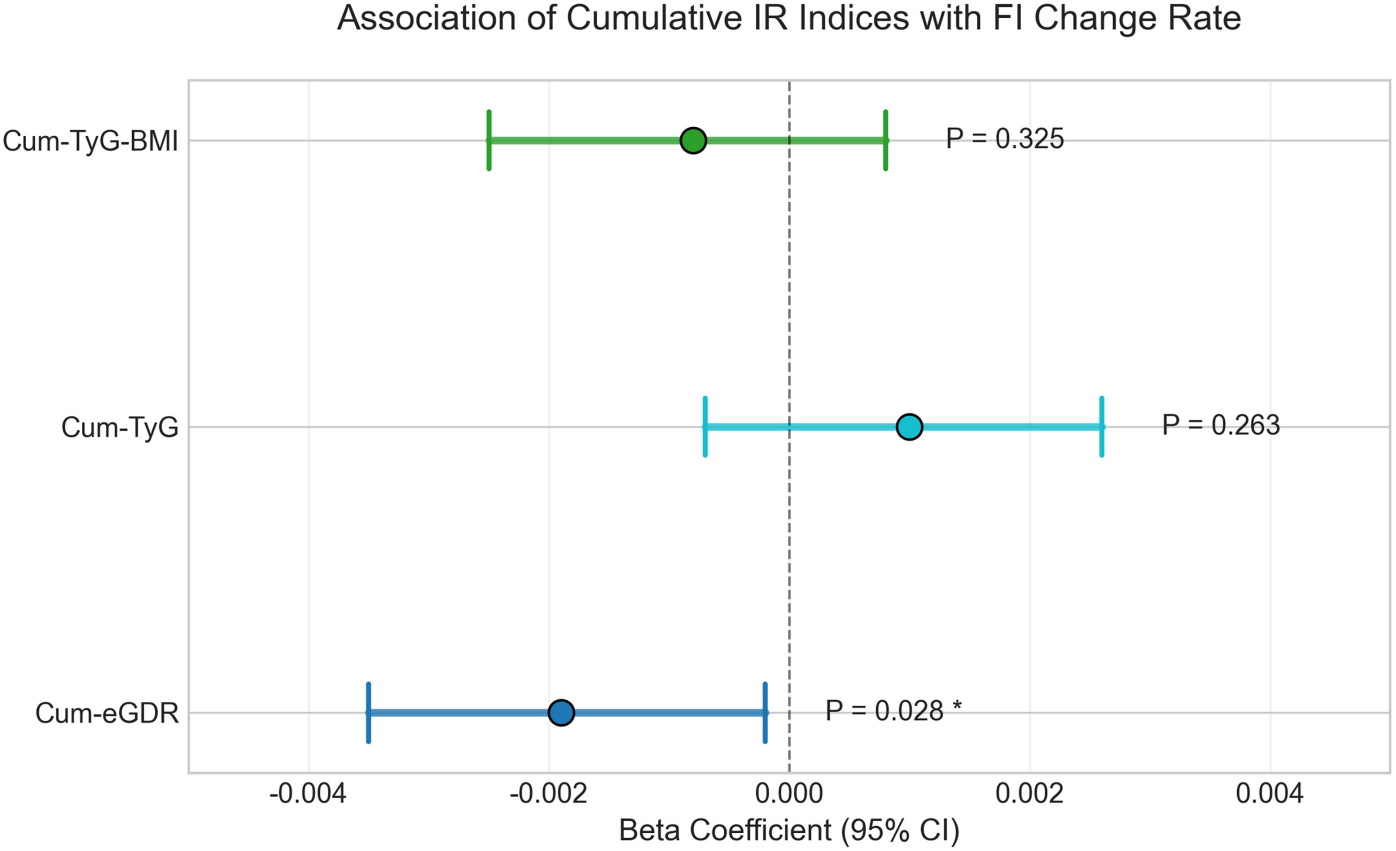
Association of cumulative IR indices with the annual rate of change in FI. Points show β, and bars the 95% CI from separate random-intercept linear mixed models with an index × time interaction (time in years since baseline). β represents the additional per-year change in FI per 1-SD higher index. Models adjust for baseline age, sex, education, rural/urban residence, smoking, drinking, cardiovascular disease (heart disease, stroke), and baseline eGDR. *N* = 451. Higher Cum-eGDR reflects greater insulin sensitivity (lower IR). An asterisk (*) indicates *P* < 0.05 for the index × time interaction in the adjusted model.

To identify the most robust predictive approach for the frailty progression trajectory, our analysis proceeded in two sequential steps. First, we compared models based on three different cumulative IR indices. The analysis revealed a critical distinction: only the interaction between Cum-eGDR and time was statistically significant [β = −0.0019, 95% CI (−0.0035, −0.0002), *P* = 0.028], whereas the interactions for Cum-TyG (*P* = 0.263) and Cum-TyG-BMI (*P* = 0.325) were not (Supplementary Table 2; Figure 2). Second, having identified Cum-eGDR as the most informative cumulative metric, we formally tested our primary hypothesis that this approach is superior to a single-point baseline assessment. As detailed in Table 2, the Cum-eGDR model showed better model fit (AIC: 1234.5 vs. 1236.8; BIC: 1289.2 vs. 1291.5) and its interaction term achieved statistical significance, unlike the baseline model (*P* = 0.028 vs. *P* = 0.134). These results support the interpretation that cumulative exposure captures frailty progression more informatively than a single baseline measure.

**TABLE 2:** Comparison of cumulative vs. baseline eGDR models for predicting FI trajectory.

Model comparison	Cum-eGDR model	Baseline eGDR model
Model fit: AIC	1234.5	1236.8
Model fit: BIC	1289.2	1291.5
Interaction β	−0.0019	−0.0012
Interaction 95% CI	[−0.0035, −0.0002]	[−0.0028, 0.0004]
Interaction *P*-value	0.028	0.134

### 3.3 Association of cumulative eGDR with frailty progression

The final adjusted LME model assessing the impact of Cum-eGDR, with detailed results presented in Table 3, revealed a significant positive main effect for time (β = 0.0065, *P* < 0.0001), indicating a significant increase in FI scores over the follow-up period after accounting for other factors. While the main effect of standardized Cum-eGDR (W1–W3) was not significant (*P* = 0.384), the key finding emerged from the interaction term.

**TABLE 3:** Linear mixed-effects model for the association between Cum-eGDR (W1–W3, per 1–SD) and FI trajectory (W1-W5).

Term	Beta (coefficient)	Std. error	*Z*-value	*P*-value	95% CI
Intercept	0.2177	0.0091	23.966	< 0.0001	(0.1999, 0.2355)
Gender (1 = male vs. 0 = female)	−0.0297	0.012	−2.487	0.0129	(−0.0532, −0.0063)
Smoking (1 = ever vs. 0 = never)	0.0032	0.0116	0.275	0.7831	(−0.0196, 0.0260)
Drinking (1 = current vs. 0 = not current)	−0.0134	0.0114	−1.173	0.2408	(−0.0358, 0.0090)
Baseline CVD (1 = yes vs. 0 = no)	0.0822	0.0119	6.911	< 0.0001	(0.0589, 0.1055)
Cum-eGDR (W1–W3, standardized)	−0.0109	0.0125	−0.871	0.384	(−0.0354, 0.0136)
Time (years since baseline)	0.0065	0.0008	7.674	< 0.0001	(0.0048, 0.0082)
Cum-eGDR (W1–W3, standardized) × time (years)	−0.0019	0.0008	−2.194	0.0282	(−0.0035, −0.0002)
Baseline age (std)	0.0217	0.0046	4.672	< 0.0001	(0.0126, 0.0308)
Baseline eGDR (std)	−0.004	0.0111	−0.359	0.7195	(−0.0258, 0.0178)

**FIGURE 3:**
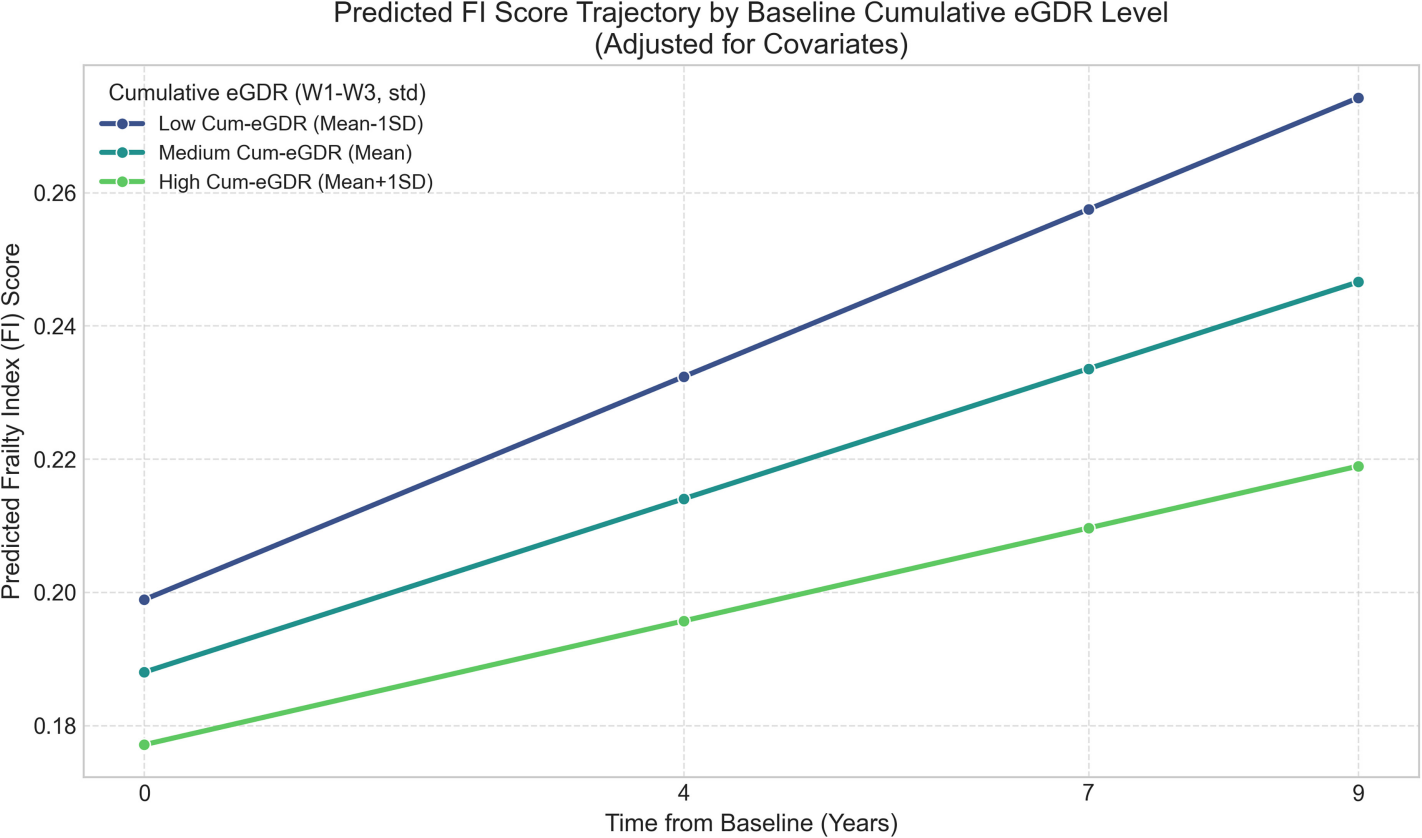
Predicted FI trajectories by baseline stratification of Cum-eGDR (W1–W3) (low: mean – 1 SD; medium: mean; high: mean + 1 SD), estimated from the adjusted random-intercept model. *N* = 451.

The interaction between standardized Cum-eGDR (W1–W3) and time (years) was statistically significant, with a coefficient of −0.0019 (95% CI: −0.0035 to −0.0002; *P* = 0.028). This significant negative interaction indicates that higher levels of Cum-eGDR (reflecting better average insulin sensitivity) were independently associated with a significantly slower rate of increase in FI scores per year over the subsequent follow-up period. Figure 3 visualizes the model-predicted FI trajectories: The slope of frailty progression is steepest for the group with low Cum-eGDR (Mean − 1SD) and flattest for the group with high Cum-eGDR (Mean + 1SD), illustrating the moderating effect of sustained insulin sensitivity on frailty progression.

### 3.4 Subgroup analyses and interactions

We conducted prespecified subgroup analyses using three-way interactions (index × time × subgroup). Detailed estimates for the Cum-eGDR × time term within each stratum are shown in Supplementary Table 3. Tests for effect modification did not identify statistically significant interactions across sex, age group, baseline FI level, CVD status, residence, or education (all P for interaction > 0.05). While point estimates generally aligned with the overall association, these null interactions should be interpreted cautiously given limited power for subgroup contrasts.

## 4 Discussion

This study, utilizing data from the nationally representative CHARLS cohort, provides longitudinal evidence on the association between cumulative IR exposure, assessed by Cum-eGDR over 4 years, and the subsequent 9-year trajectory of FI changes among middle-aged and older Chinese adults with diabetes. Our key finding is that higher Cum-eGDR, indicative of better-maintained average insulin sensitivity, is independently associated with a statistically slower rate of frailty progression. Over 9 years, a 1-SD higher Cum-eGDR corresponded to an estimated ≈0.017 lower FI (β × years = 0.0019 × 9), which is modest in absolute magnitude but represents ≈29% of the average yearly FI increase (0.0019/0.0065). Furthermore, when compared with Cum-TyG and Cum-TyG-BMI, Cum-eGDR demonstrated comparatively superior predictive value for the FI trajectory in this population. Subgroup patterns were broadly similar across sex, age, baseline frailty, baseline CVD status, residence, and education; however, the absence of statistically significant interactions should be interpreted cautiously given limited power for subgroup contrasts.

Our central finding supports the interpretation of metabolic health as a cornerstone of healthy aging and specifically highlights the importance of sustained insulin sensitivity in being associated with a slower pace of the progression of frailty, a major geriatric syndrome. This aligns with previous prospective studies identifying reduced insulin sensitivity as an independent predictor of frailty development. By moving beyond a single baseline IR assessment, our study employed a cumulative exposure metric, which with two time points functions as a time-weighted average and arguably may better capture the longer-term impact of long-term metabolic dysregulation on functional decline. The observed superiority of Cum-eGDR over Cum-TyG and Cum-TyG-BMI in predicting the frailty trajectory slope might stem from its unique composition. The eGDR calculation integrates not only glycemic control (HbA1c) but also central obesity (waist circumference) and blood pressure – key components of the broader cardiometabolic risk profile (20, 21). Waist circumference serves as a proxy for visceral adiposity, closely linked to pro-inflammatory cytokine secretion, while hypertension signifies vascular dysfunction. Thus, Cum-eGDR may encapsulate a more comprehensive picture of the interplay between classic glucocentric IR, obesity-driven inflammation, and vascular health status, all critical upstream drivers of frailty. In contrast, TyG and TyG-BMI, primarily reflecting hepatic IR and lipid-glucose interactions (22), might less effectively capture this multi-system dysregulation pertinent to frailty progression in our cohort.

Elucidating the potential biological mechanisms linking Cum-eGDR to frailty progression requires consideration at cellular and molecular levels. IR fundamentally involves impaired insulin signaling in target tissues (23). In skeletal muscle, a primary site for glucose disposal and crucial for physical function, this leads to impaired glucose uptake and utilization, potentially causing bioenergetic deficits and mitochondrial dysfunction (24). Concurrently, attenuated insulin signaling, as an anabolic hormone, hinders muscle protein synthesis (MPS) and may promote catabolism (25), contributing to sarcopenia, a core component of frailty (26). Furthermore, the milieu of chronic low-grade inflammation (“inflammaging”) and oxidative stress often accompanying IR, along with associated endothelial dysfunction (27–29) (which impairs muscle microcirculation, critical in pathologies like PAD), collectively creates a complex network driving multi-system decline. Consistent with these mechanisms, our observational findings are compatible with the hypothesis that maintaining better insulin sensitivity may attenuate pathways linked to frailty progression. However, these observational findings do not prove this hypothesis. Regarding subgroups, our null interaction tests, such as the finding for baseline frailty where *P* = 0.087, may reflect limited power rather than an equivalence of effects. Against the backdrop of diabetes as a major frailty risk factor, insulin sensitivity could plausibly represent a broadly relevant correlate of slower frailty progression. A bidirectional relationship between frailty and metabolic dysregulation may also contribute, whereby IR promotes frailty, and frailty-related sarcopenia or inactivity worsens IR (30). Although some studies suggest age-specific IR–frailty links, our trajectory analysis did not confirm age heterogeneity (31), differences in design, populations, or outcomes may explain these discrepancies.

Our findings hold potential clinical and public health implications. First, they are consistent with a holistic “cardiometabolic health” perspective that extends beyond glycemic control to include insulin sensitivity, central adiposity, and blood pressure, with the goal of delaying frailty. Secondly, Cum-eGDR, as a relatively accessible index, may help monitor metabolic status and identify individuals at higher long-term frailty risk, recognizing that the absolute effect size is modest. For vascular surgery, these results are particularly pertinent. Frailty markedly increases perioperative risks in diabetic patients undergoing vascular procedures. Incorporating an assessment of recent metabolic trajectory alongside standard frailty screening during preoperative evaluation could offer richer prognostic information regarding surgical tolerance and functional recovery. For high-risk individuals identified with low Cum-eGDR, prehabilitation approaches targeting insulin sensitivity, including exercise, nutrition, and medication optimization, merit evaluation in future studies.

The strengths of this study include its prospective design using a nationally sampled cohort, a long follow-up period (up to 9 years), longitudinal trajectory analysis of frailty (LME models), assessment of cumulative exposure, comparison of different IR indices, and examination of multiple potential effect modifiers. However, certain limitations must be acknowledged: (1) The observational design precludes definitive causal inference, and residual confounding remains possible from unmeasured factors such as physical activity, diet, and specific diabetes medications, which were not fully captured or adjusted. (2) The FI construction relies partly on self-reported data and, while multi-domain, might not capture all facets of frailty and includes fewer items than the often-recommended 30–40. (3) Cumulative indices were estimated from two time points (W1 and W3) using a trapezoidal approach that, with two observations, is equivalent to a time-weighted average; it does not capture within-interval dynamics and likely biases associations toward the null. (4) The final sample size of 451 participants, resulting from strict inclusion criteria, may limit statistical power for detecting weaker subgroup interactions and raises the possibility of selection bias; the analytic sample is a highly selected subset of the baseline cohort, so generalizability is primarily to similar participants with complete repeated measures. (5) Findings are specific to individuals with baseline diabetes and may not be generalizable to non-diabetic or prediabetic populations. (6) Multiple hypothesis testing across models and subgroups increases the chance of Type I error; we report exact *P*-values, did not apply formal multiplicity corrections given correlated tests, and interpret subgroup findings cautiously.

Future research should aim to utilize more frequent biomarker data to construct more refined IR exposure trajectory models; employ methods like Mendelian randomization to probe causality; validate findings in diverse populations; explore the mediating roles of specific pathways like inflammation, sarcopenia markers, or even the gut microbiome; and ultimately conduct randomized controlled trials to evaluate the efficacy of interventions targeting insulin sensitivity improvement on delaying frailty progression. In surgical populations, trials of prehabilitation strategies that improve insulin sensitivity and test effects on perioperative frailty trajectories are also warranted.

## 5 Conclusion

In conclusion, this study provides novel longitudinal evidence of a statistically significant association between higher long-term insulin sensitivity, as assessed by Cum-eGDR, and a slower rate of frailty progression among middle-aged and older Chinese adults with diabetes. While modest in magnitude, Cum-eGDR showed better model fit than Cum-TyG and Cum-TyG-BMI and associations were generally similar across major subgroups without statistically significant interactions. While these findings support the use of Cum-eGDR for risk assessment and monitoring, whether improving insulin sensitivity can alter frailty trajectories requires confirmation in interventional studies, and generalizability is primarily to similar participants with repeated measures.

## Data Availability

The datasets analyzed for this study are available in the China Health and Retirement Longitudinal Study (CHARLS) repository. This data can be accessed at: http://charls.pku.edu.cn/. Access to the data requires registration and application via the CHARLS website. Further inquiries can be directed to the corresponding author.
