# Supplementary material for: Cumulative estimated glucose disposal rate predicts frailty progression in Chinese adults with diabetes: a 9-year follow-up cohort study
Source: Front Med (Lausanne). 2025 Dec 22;12:1617556. doi: 10.3389/fmed.2025.1617556 (PMC12767217; doi:10.3389/fmed.2025.1617556)
Supplement: Supplementary file 1 [file Supplementary_file_1.docx]

**Supplementary Table S1.** FI components (26 items) and coding rules.

| **Domain** | **Variable(s) (CHARLS)** | **Deficit Definition (Coding=1)** | **Notes / Coding Details** |
| --- | --- | --- | --- |
| Chronic Diseases | hibpe(W1) | Has hypertension | Self-report/meds/measurement |
| Chronic Diseases | dyslipe | Has dyslipidemia | Self-reported |
| Chronic Diseases | diabe(W1) | Has diabetes | Self-report/meds/biomarker |
| Chronic Diseases | cancre | Has cancer | Self-reported |
| Chronic Diseases | lunge | Chronic lung disease | Self-reported |
| Chronic Diseases | hearte | Heart disease | Self-reported |
| Chronic Diseases | stroke | Stroke | Self-reported |
| Chronic Diseases | psyche | Psychiatric problem | Self-reported |
| Chronic Diseases | arthe | Arthritis/rheumatism | Self-reported |
| Chronic Diseases | liver | Liver disease | Self-reported |
| Chronic Diseases | kidneye | Kidney disease | Self-reported |
| Chronic Diseases | digeste | Stomach/digestive disease | Self-reported |
| Chronic Diseases | asthmae | Asthma | Self-reported |
| Symptoms/Sensory | srh | Self-rated health Fair/Poor (≥4) | |
| Symptoms/Sensory | eyesight_distance | Difficulty near/far vision (≥3) | Derived |
| Symptoms/Sensory | hear | Difficulty hearing (≥4) | Derived |
| Symptoms/Sensory | fall_down | Fallen down in last year/two years (=1) | |
| Symptoms/Sensory | da0425i–da0425i15 | Pain in ≥3 body parts | Derived; count where value=1 |
| Symptoms/Sensory | sleep | Sleep <5h or >9h |  |
| Symptoms/Sensory | teeth | Teeth problems / lost teeth (=1) | |
| Symptoms/Sensory | satisfie | Dissatisfied/Very dissatisfied (≥4) | |
| Function (ADL/IADL) | adlab_c | ≥1 ADL difficulty (>0) |  |
| Function (ADL/IADL) | iadl | ≥1 IADL difficulty (>0) |  |
| Cognition/Mood | total_cognition | Cognitive impairment (<10) | 0–21 scale (lower=worse) |

**Supplementary Table S2.** Comparison of associations between different cumulative IR indices and the annual rate of change in FI score (index×time interaction), with full coefficients.

| **Cumulative Index** | **Beta for Interaction** | **95% CI for Interaction** | **P-value** |
| --- | --- | --- | --- |
| Cum-eGDR | -0.0019 | [-0.0035, -0.0002] | 0.028 * |
| Cum-TyG | 0.001 | [-0.0007, 0.0026] | 0.263 |
| Cum-TyG-BMI | -0.0008 | [-0.0025, 0.0008] | 0.325 |

**Supplementary Table S3.** Subgroup analyses of Cum-eGDR with FI change (index×time β) with three-way interaction P-values.

| **Subgroup** | **Category** | **Beta** | **95% CI** | **P-value** | **person-waves** | **Three-way P** |
| --- | --- | --- | --- | --- | --- | --- |
| Gender | Female | -0.0015 | [-0.0040, 0.0010] | 0.2533 | 639 | 0.596 |
| Gender | Male | -0.0024 | [-0.0046, -0.0001] | 0.0371 | 714 | 0.5969 |
| Age-Group | <65 | -0.0015 | [-0.0033, 0.0004] | 0.1181 | 1131 | 0.2488 |
| Age-Group | >=65 | -0.004 | [-0.0081, 0.0001] | 0.0569 | 222 | 0.2488 |
| Baseline-FI | <0.1 | 0.0052 | [-0.0038, 0.0141] | 0.2577 | 81 | 0.0873 |
| Baseline-FI | >=0.1 | -0.0022 | [-0.0038, -0.0005] | 0.0131 | 1272 | 0.0873 |
| Baseline-CVD | No | -0.0015 | [-0.0033, 0.0003] | 0.1122 | 1092 | 0.987 |
| Baseline-CVD | Yes | -0.0015 | [-0.0060, 0.0030] | 0.5116 | 261 | 0.987 |
| Residence | Rural | -0.0014 | [-0.0035, 0.0008] | 0.2124 | 780 | 0.6186 |
| Residence | Urban | -0.0023 | [-0.0049, 0.0004] | 0.095 | 573 | 0.6186 |
| Education | <=Middle | -0.0019 | [-0.0036, -0.0001] | 0.0357 | 1188 | 0.9343 |
| Education | >Middle | -0.0016 | [-0.0069, 0.0036] | 0.5397 | 165 | 0.9343 |
